# Supplementary material for: Structure of KAP1 tripartite motif identifies molecular interfaces required for retroelement silencing
Source: Proc Natl Acad Sci U S A. 2019 Jul 9;116(30):15042–51. doi: 10.1073/pnas.1901318116 (PMC6660772; doi:10.1073/pnas.1901318116)
Supplement: Supplementary File [file pnas.1901318116.sd01.pdf]

Full wwPDB X-ray Structure Validation Report (i)

Dec 19, 2018 – 01:59 PM GMT

PDB ID	:	6QAJ
Title	:	Structure of the tripartite motif of KAP1/TRIM28
Deposited on	:	2018-12-19
Resolution	:	2.90 Å(reported)

This is a Full wwPDB X-ray Structure Validation Report.

This report is produced by the wwPDB biocuration pipeline after annotation of the structure.

We welcome your comments at *validation@mail.wwpdb.org*
A user guide is available at
https://www.wwpdb.org/validation/2017/XrayValidationReportHelp
with specific help available everywhere you see the (i) symbol.

CONFIDENTIAL VALIDATION REPORT

The following versions of software and data (see references (i)) were used in the production of this report:

MolProbity	:	4.02b-467
Xtriage (Phenix)	:	1.13
EDS	:	rb-20031633
Percentile statistics	:	20171227.v01 (using entries in the PDB archive December 27th 2017)
Refmac	:	5.8.0158
CCP4	:	7.0 (Gargrove)
Ideal geometry (proteins)	:	Engh & Huber (2001)
Ideal geometry (DNA, RNA)	:	Parkinson et al. (1996)
Validation Pipeline (wwPDB-VP)	:	rb-20031633

1 Overall quality at a glance (i)

The following experimental techniques were used to determine the structure:
X-RAY DIFFRACTION

The reported resolution of this entry is 2.90 Å.

Percentile scores (ranging between 0-100) for global validation metrics of the entry are shown in the following graphic. The table shows the number of entries on which the scores are based.

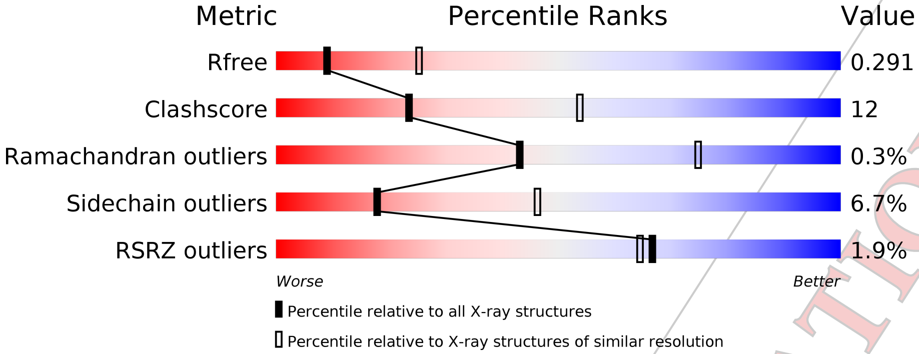

Metric	Whole archive (#Entries)	Similar resolution (#Entries, resolution range(Å))
R_{free}	111664	1716 (2.90-2.90)
Clashscore	122126	1924 (2.90-2.90)
Ramachandran outliers	120053	1884 (2.90-2.90)
Sidechain outliers	120020	1886 (2.90-2.90)
RSRZ outliers	108989	1669 (2.90-2.90)

The table below summarises the geometric issues observed across the polymeric chains and their fit to the electron density. The red, orange, yellow and green segments on the lower bar indicate the fraction of residues that contain outliers for >=3, 2, 1 and 0 types of geometric quality criteria. A grey segment represents the fraction of residues that are not modelled. The numeric value for each fraction is indicated below the corresponding segment, with a dot representing fractions <=5% The upper red bar (where present) indicates the fraction of residues that have poor fit to the electron density. The numeric value is given above the bar.

Mol	Chain	Length	Quality of chain
1	A	544	2% / 59% 19% • 19%
1	B	544	% / 61% 19% • 19%

CONFIDENTIAL VALIDATION REPORT

2 Entry composition (i)

There are 2 unique types of molecules in this entry. The entry contains 13867 atoms, of which 6904 are hydrogens and 0 are deuteriums.

In the tables below, the ZeroOcc column contains the number of atoms modelled with zero occupancy, the AltConf column contains the number of residues with at least one atom in alternate conformation and the Trace column contains the number of residues modelled with at most 2 atoms.

- Molecule 1 is a protein called Endolysin,Transcription intermediary factor 1-beta.

Mol	Chain	Residues	Atoms						ZeroOcc	AltConf	Trace
			Total	C	H	N	O	S			
1	A	439	6891	2166	3424	633	644	24	0	0	0
1	B	441	6968	2179	3480	644	640	25	0	0	0

There are 52 discrepancies between the modelled and reference sequences:

Chain	Residue	Modelled	Actual	Comment	Reference
A	-130	MET	-	initiating methionine	UNP P00720
A	-129	GLY	-	expression tag	UNP P00720
A	-128	SER	-	expression tag	UNP P00720
A	-127	SER	-	expression tag	UNP P00720
A	-126	HIS	-	expression tag	UNP P00720
A	-125	HIS	-	expression tag	UNP P00720
A	-124	HIS	-	expression tag	UNP P00720
A	-123	HIS	-	expression tag	UNP P00720
A	-122	HIS	-	expression tag	UNP P00720
A	-121	HIS	-	expression tag	UNP P00720
A	-120	SER	-	expression tag	UNP P00720
A	-119	GLN	-	expression tag	UNP P00720
A	-118	ASP	-	expression tag	UNP P00720
A	-117	PRO	-	expression tag	UNP P00720
A	-116	ASN	-	expression tag	UNP P00720
A	-115	SER	-	expression tag	UNP P00720
A	-114	SER	-	expression tag	UNP P00720
A	-113	SER	-	expression tag	UNP P00720
A	-112	GLU	-	expression tag	UNP P00720
A	-111	ASN	-	expression tag	UNP P00720
A	-110	LEU	-	expression tag	UNP P00720
A	-109	TYR	-	expression tag	UNP P00720
A	-108	PHE	-	expression tag	UNP P00720
A	-107	GLN	-	expression tag	UNP P00720
A	-106	GLY	-	expression tag	UNP P00720

Continued on next page...

CONFIDENTIAL VALIDATION REPORT

Continued from previous page...

Chain	Residue	Modelled	Actual	Comment	Reference
A	55	ALA	-	linker	UNP P00720
B	-130	MET	-	initiating methionine	UNP P00720
B	-129	GLY	-	expression tag	UNP P00720
B	-128	SER	-	expression tag	UNP P00720
B	-127	SER	-	expression tag	UNP P00720
B	-126	HIS	-	expression tag	UNP P00720
B	-125	HIS	-	expression tag	UNP P00720
B	-124	HIS	-	expression tag	UNP P00720
B	-123	HIS	-	expression tag	UNP P00720
B	-122	HIS	-	expression tag	UNP P00720
B	-121	HIS	-	expression tag	UNP P00720
B	-120	SER	-	expression tag	UNP P00720
B	-119	GLN	-	expression tag	UNP P00720
B	-118	ASP	-	expression tag	UNP P00720
B	-117	PRO	-	expression tag	UNP P00720
B	-116	ASN	-	expression tag	UNP P00720
B	-115	SER	-	expression tag	UNP P00720
B	-114	SER	-	expression tag	UNP P00720
B	-113	SER	-	expression tag	UNP P00720
B	-112	GLU	-	expression tag	UNP P00720
B	-111	ASN	-	expression tag	UNP P00720
B	-110	LEU	-	expression tag	UNP P00720
B	-109	TYR	-	expression tag	UNP P00720
B	-108	PHE	-	expression tag	UNP P00720
B	-107	GLN	-	expression tag	UNP P00720
B	-106	GLY	-	expression tag	UNP P00720
B	55	ALA	-	linker	UNP P00720

- Molecule 2 is ZINC ION (three-letter code: ZN) (formula: Zn).

Mol	Chain	Residues	Atoms	ZeroOcc	AltConf
2	B	4	Total Zn<br>4 4	0	0
2	A	4	Total Zn<br>4 4	0	0

CONFIDENTIAL VALIDATION REPORT

3 Residue-property plots (i)

These plots are drawn for all protein, RNA and DNA chains in the entry. The first graphic for a chain summarises the proportions of the various outlier classes displayed in the second graphic. The second graphic shows the sequence view annotated by issues in geometryand electron density. Residues are color-coded according to the number of geometric quality criteria for which they contain at least one outlier: green = 0, yellow = 1, orange = 2 and red = 3 or more. A red dot above a residue indicates a poor fit to the electron density (RSRZ > 2). Stretches of 2 or more consecutive residues without any outlier are shown as a green connector. Residues present in the sample, but not in the model, are shown in grey.

- Molecule 1: Endolysin,Transcription intermediary factor 1-beta

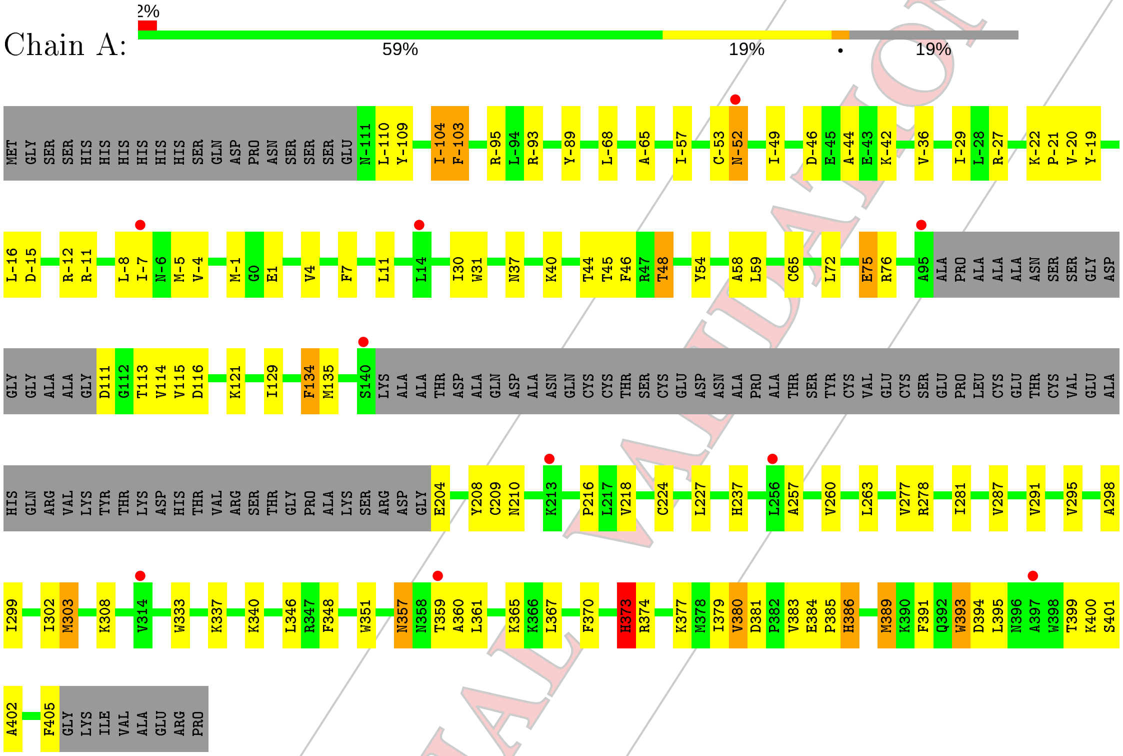

- Molecule 1: Endolysin,Transcription intermediary factor 1-beta

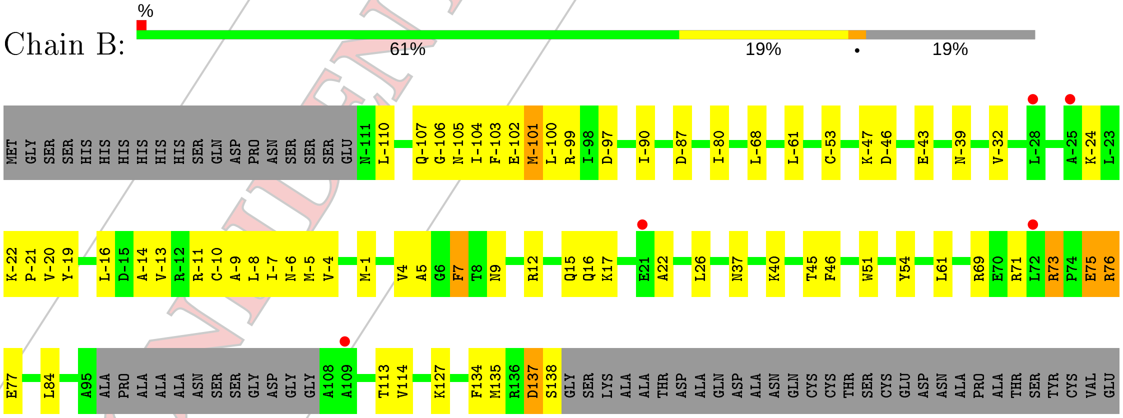

CONFIDENTIAL VALIDATION REPORT

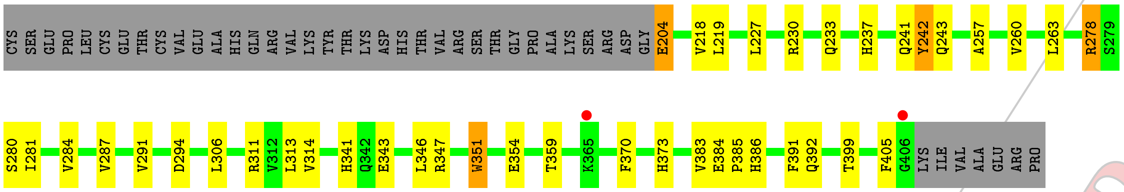

CONFIDENTIAL VALIDATION REPORT

4 Data and refinement statistics (i)

Property	Value	Source
Space group	C 2 2 21	Depositor
Cell constants<br>a, b, c, α, β, γ	59.77Å 169.33Å 374.51Å<br>90.00° 90.00° 90.00°	Depositor
Resolution (Å)	62.80 – 2.90<br>93.63 – 2.63	Depositor<br>EDS
% Data completeness<br>(in resolution range)	41.4 (62.80-2.90)<br>29.6 (93.63-2.63)	Depositor<br>EDS
R_{merge}	0.08	Depositor
R_{sym}	(Not available)	Depositor
$< I/\sigma(I) >$ [1]	0.33 (at 2.62Å)	Xtriage
Refinement program	PHENIX 1.14-3260	Depositor
R, R_{free}	0.261 , 0.291<br>0.261 , 0.291	Depositor<br>DCC
R_{free} test set	896 reflections (4.87%)	wwPDB-VP
Wilson B-factor (Å^2)	76.1	Xtriage
Anisotropy	0.178	Xtriage
Bulk solvent k_{sol}(e/Å^3), B_{sol}(Å^2)	0.32 , 44.6	EDS
L-test for twinning[2]	$< \|L\| > = 0.48$, $< L^2 > = 0.31$	Xtriage
Estimated twinning fraction	No twinning to report.	Xtriage
F_o,F_c correlation	0.88	EDS
Total number of atoms	13867	wwPDB-VP
Average B, all atoms (Å^2)	121.0	wwPDB-VP

Xtriage's analysis on translational NCS is as follows: *The largest off-origin peak in the Patterson function is 5.13% of the height of the origin peak. No significant pseudotranslation is detected.*

[1] Intensities estimated from amplitudes.

[2] Theoretical values of $< |L| >$, $< L^2 >$ for acentric reflections are 0.5, 0.333 respectively for untwinned datasets, and 0.375, 0.2 for perfectly twinned datasets.

CONFIDENTIAL VALIDATION REPORT

5 Model quality (i)

5.1 Standard geometry (i)

Bond lengths and bond angles in the following residue types are not validated in this section: ZN

The Z score for a bond length (or angle) is the number of standard deviations the observed value is removed from the expected value. A bond length (or angle) with $|Z| > 5$ is considered an outlier worth inspection. RMSZ is the root-mean-square of all Z scores of the bond lengths (or angles).

Mol	Chain	Bond lengths		Bond angles	
		RMSZ	$\#\|Z\| >5$	RMSZ	$\#\|Z\| >5$
1	A	0.34	0/3523	0.52	1/4755 (0.0%)
1	B	0.33	0/3543	0.59	3/4775 (0.1%)
All	All	0.33	0/7066	0.56	4/9530 (0.0%)

Chiral center outliers are detected by calculating the chiral volume of a chiral center and verifying if the center is modelled as a planar moiety or with the opposite hand.A planarity outlier is detected by checking planarity of atoms in a peptide group, atoms in a mainchain group or atoms of a sidechain that are expected to be planar.

Mol	Chain	#Chirality outliers	#Planarity outliers
1	A	0	3

There are no bond length outliers.

All (4) bond angle outliers are listed below:

Mol	Chain	Res	Type	Atoms	Z	Observed(^{o})	Ideal(^{o})
1	B	73	ARG	NE-CZ-NH1	-11.15	114.72	120.30
1	B	73	ARG	NE-CZ-NH2	9.12	124.86	120.30
1	B	-101	MET	CG-SD-CE	6.76	111.01	100.20
1	A	-42	LYS	CD-CE-NZ	5.09	123.41	111.70

There are no chirality outliers.

All (3) planarity outliers are listed below:

Mol	Chain	Res	Type	Group
1	A	373	HIS	Peptide
1	A	374	ARG	Peptide
1	A	75	GLU	Peptide

CONFIDENTIAL VALIDATION REPORT

5.2 Too-close contacts (i)

In the following table, the Non-H and H(model) columns list the number of non-hydrogen atoms and hydrogen atoms in the chain respectively. The H(added) column lists the number of hydrogen atoms added and optimized by MolProbity. The Clashes column lists the number of clashes within the asymmetric unit, whereas Symm-Clashes lists symmetry related clashes.

Mol	Chain	Non-H	H(model)	H(added)	Clashes	Symm-Clashes
1	A	3467	3424	3426	90	0
1	B	3488	3480	3483	109	0
2	A	4	0	0	0	0
2	B	4	0	0	0	0
All	All	6963	6904	6909	168	0

The all-atom clashscore is defined as the number of clashes found per 1000 atoms (including hydrogen atoms). The all-atom clashscore for this structure is 12.

All (168) close contacts within the same asymmetric unit are listed below, sorted by their clash magnitude.

Atom-1	Atom-2	Interatomic distance (Å)	Clash overlap (Å)
1:B:-101:MET:HE2	1:B:-6:ASN:HB2	1.39	1.05
1:B:-14:ALA:O	1:B:-10:CYS:SG	2.29	0.91
1:B:9:ASN:O	1:B:12:ARG:HG2	1.76	0.85
1:A:-12:ARG:NH1	1:A:46:PHE:O	2.10	0.84
1:A:299:ILE:HG23	1:B:306:LEU:HD23	1.63	0.80
1:B:-90:ILE:HG22	1:B:-80:ILE:HD12	1.68	0.76
1:B:-13:VAL:O	1:B:-10:CYS:HB2	1.87	0.75
1:B:-4:VAL:HG22	1:B:4:VAL:HG11	1.69	0.73
1:B:-104:ILE:O	1:B:-104:ILE:HG12	1.88	0.71
1:A:75:GLU:O	1:A:76:ARG:HB2	1.90	0.70
1:B:-90:ILE:HD12	1:B:-68:LEU:HD11	1.72	0.69
1:A:-20:VAL:O	1:A:-16:LEU:HD13	1.93	0.69
1:B:-104:ILE:O	1:B:-104:ILE:CG1	2.40	0.68
1:A:204:GLU:OE1	1:A:204:GLU:N	2.27	0.68
1:B:-105:ASN:OD1	1:B:-104:ILE:N	2.25	0.68
1:A:287:VAL:O	1:A:291:VAL:HG23	1.93	0.68
1:B:-101:MET:HE1	1:B:-9:ALA:HA	1.75	0.67
1:A:295:VAL:HG22	1:B:386:HIS:ND1	2.09	0.67
1:B:-101:MET:SD	1:B:51:TRP:HZ3	2.18	0.66
1:B:9:ASN:HA	1:B:12:ARG:HG2	1.78	0.65
1:A:291:VAL:HG22	1:B:384:GLU:HA	1.78	0.65
1:A:302:ILE:HD11	1:B:306:LEU:HD22	1.79	0.65
1:B:45:THR:HG21	1:B:54:TYR:CE2	2.32	0.65

Continued on next page...

CONFIDENTIAL VALIDATION REPORT

Continued from previous page...

Atom-1	Atom-2	Interatomic distance (Å)	Clash overlap (Å)
1:B:-101:MET:HE2	1:B:-6:ASN:CB	2.23	0.64
1:B:-20:VAL:O	1:B:-16:LEU:HD13	1.97	0.64
1:B:-20:VAL:HG22	1:B:15:GLN:HG3	1.80	0.64
1:B:-104:ILE:HD12	1:B:-10:CYS:HA	1.79	0.63
1:B:-101:MET:CE	1:B:-6:ASN:HB2	2.25	0.61
1:B:278:ARG:HE	1:B:281:ILE:HD11	1.67	0.59
1:B:9:ASN:C	1:B:12:ARG:HG2	2.22	0.59
1:A:37:ASN:O	1:A:40:LYS:HG2	2.03	0.58
1:A:72:LEU:HB2	1:A:135:MET:SD	2.43	0.58
1:B:45:THR:HG21	1:B:54:TYR:HE2	1.69	0.57
1:B:-107:GLN:HG2	1:B:51:TRP:HB3	1.85	0.57
1:A:44:THR:O	1:A:48:THR:OG1	2.21	0.57
1:A:302:ILE:HD11	1:B:306:LEU:CD2	2.35	0.57
1:B:9:ASN:CA	1:B:12:ARG:HG2	2.34	0.57
1:B:-5:MET:SD	1:B:46:PHE:HE2	2.27	0.57
1:B:-90:ILE:CG2	1:B:-80:ILE:HD12	2.35	0.57
1:A:-19:TYR:HD1	1:A:-8:LEU:HD23	1.69	0.57
1:A:402:ALA:HB3	1:B:386:HIS:O	2.05	0.56
1:A:348:PHE:HB3	1:B:84:LEU:HD23	1.87	0.56
1:A:401:SER:HB3	1:B:313:LEU:HD22	1.88	0.55
1:B:384:GLU:N	1:B:385:PRO:CD	2.70	0.55
1:B:242:TYR:O	1:B:243:GLN:HG2	2.07	0.54
1:A:298:ALA:O	1:A:302:ILE:HG23	2.08	0.54
1:A:-95:ARG:HH11	1:A:-95:ARG:HG3	1.72	0.54
1:A:351:TRP:HH2	1:B:135:MET:HE1	1.72	0.54
1:B:61:LEU:HD11	1:B:69:ARG:HH11	1.72	0.54
1:B:-101:MET:SD	1:B:51:TRP:CZ3	3.00	0.53
1:B:137:ASP:O	1:B:138:SER:HB2	2.08	0.53
1:B:-32:VAL:HG22	1:B:-7:ILE:HD13	1.88	0.53
1:A:386:HIS:NE2	1:B:294:ASP:OD2	2.42	0.53
1:B:37:ASN:O	1:B:40:LYS:HG2	2.08	0.53
1:B:383:VAL:HG23	1:B:383:VAL:O	2.08	0.53
1:A:65:CYS:HB3	1:A:72:LEU:HD21	1.89	0.53
1:B:113:THR:O	1:B:113:THR:HG22	2.09	0.53
1:B:237:HIS:N	1:B:237:HIS:CD2	2.77	0.52
1:B:71:ARG:HE	1:B:73:ARG:NH1	2.08	0.52
1:A:218:VAL:O	1:A:218:VAL:HG12	2.09	0.52
1:A:224:CYS:SG	1:A:237:HIS:HE1	2.32	0.52
1:B:76:ARG:HG2	1:B:76:ARG:O	2.10	0.51
1:A:-110:LEU:HD12	1:A:59:LEU:HD12	1.92	0.51
1:B:-19:TYR:HD1	1:B:-8:LEU:HD23	1.74	0.51

Continued on next page...

CONFIDENTIAL VALIDATION REPORT

Continued from previous page...

Atom-1	Atom-2	Interatomic distance (Å)	Clash overlap (Å)
1:A:-109:TYR:HD1	1:A:-109:TYR:O	1.93	0.51
1:A:361:LEU:HD22	1:B:134:PHE:HE2	1.76	0.51
1:A:-109:TYR:CD1	1:A:-109:TYR:O	2.64	0.51
1:B:-5:MET:O	1:B:-1:MET:HG2	2.11	0.51
1:B:281:ILE:O	1:B:284:VAL:HG22	2.11	0.51
1:B:73:ARG:O	1:B:75:GLU:N	2.44	0.50
1:A:-46:ASP:OD1	1:A:-46:ASP:N	2.42	0.50
1:B:73:ARG:HH11	1:B:73:ARG:HG2	1.75	0.50
1:A:346:LEU:HD21	1:B:263:LEU:HB3	1.94	0.50
1:A:263:LEU:HD22	1:B:346:LEU:HD21	1.94	0.50
1:A:-104:ILE:H	1:A:-104:ILE:HD13	1.77	0.49
1:A:340:LYS:HE3	1:B:-106:GLY:HA3	1.94	0.49
1:A:-68:LEU:HD12	1:A:-65:ALA:HB3	1.95	0.49
1:A:30:ILE:HG13	1:A:31:TRP:N	2.26	0.49
1:B:37:ASN:N	1:B:37:ASN:OD1	2.45	0.49
1:A:37:ASN:OD1	1:A:37:ASN:N	2.45	0.49
1:A:295:VAL:HG22	1:B:386:HIS:CG	2.48	0.49
1:A:7:PHE:O	1:A:11:LEU:HG	2.12	0.49
1:B:-22:LYS:N	1:B:-21:PRO:HD2	2.28	0.49
1:B:-46:ASP:OD1	1:B:-46:ASP:N	2.46	0.49
1:A:361:LEU:HD22	1:B:134:PHE:CE2	2.48	0.48
1:A:-49:ILE:HD12	1:A:-44:ALA:HB2	1.96	0.48
1:A:-22:LYS:N	1:A:-21:PRO:HD2	2.28	0.48
1:A:135:MET:HE2	1:B:351:TRP:HH2	1.79	0.48
1:A:-4:VAL:HG22	1:A:4:VAL:HG11	1.95	0.48
1:A:-52:ASN:O	1:A:-52:ASN:ND2	2.32	0.48
1:B:219:LEU:HD13	1:B:233:GLN:OE1	2.13	0.48
1:A:-68:LEU:O	1:A:-65:ALA:N	2.47	0.48
1:B:22:ALA:O	1:B:26:LEU:HG	2.14	0.47
1:B:-24:LYS:HD2	1:B:5:ALA:HB1	1.96	0.47
1:A:-93:ARG:HD2	1:A:-89:TYR:CE1	2.50	0.47
1:A:113:THR:O	1:A:113:THR:HG22	2.15	0.47
1:A:295:VAL:HG22	1:B:386:HIS:CE1	2.49	0.47
1:B:287:VAL:O	1:B:291:VAL:HG23	2.14	0.47
1:A:405:PHE:HA	1:B:383:VAL:HG12	1.97	0.47
1:B:71:ARG:HE	1:B:73:ARG:HH12	1.62	0.47
1:B:9:ASN:O	1:B:12:ARG:CG	2.56	0.47
1:A:405:PHE:CB	1:B:383:VAL:HG12	2.46	0.46
1:A:134:PHE:HE1	1:B:354:GLU:HB2	1.80	0.46
1:B:-102:GLU:O	1:B:-99:ARG:N	2.48	0.46
1:A:227:LEU:HD12	1:B:359:THR:HB	1.97	0.46

Continued on next page...

CONFIDENTIAL VALIDATION REPORT

Continued from previous page...

Atom-1	Atom-2	Interatomic distance (Å)	Clash overlap (Å)
1:A:361:LEU:HD23	1:A:365:LYS:HE2	1.97	0.46
1:A:45:THR:HG21	1:A:54:TYR:CE1	2.51	0.46
1:A:399:THR:O	1:A:399:THR:HG23	2.16	0.45
1:B:9:ASN:HA	1:B:12:ARG:CG	2.44	0.45
1:A:-104:ILE:HG12	1:A:-103:PHE:N	2.31	0.45
1:B:45:THR:HG21	1:B:54:TYR:CD2	2.52	0.45
1:B:7:PHE:N	1:B:7:PHE:CD1	2.84	0.45
1:A:-57:ILE:HG21	1:A:-53:CYS:SG	2.56	0.45
1:A:351:TRP:CH2	1:B:135:MET:HE1	2.50	0.45
1:B:280:SER:O	1:B:284:VAL:HG13	2.17	0.45
1:B:-10:CYS:HB3	1:B:51:TRP:HH2	1.81	0.45
1:B:-61:LEU:HD21	1:B:-53:CYS:HB2	1.99	0.45
1:A:401:SER:CB	1:B:313:LEU:HD22	2.47	0.45
1:B:278:ARG:O	1:B:281:ILE:HG12	2.16	0.45
1:A:135:MET:CE	1:B:351:TRP:HH2	2.30	0.45
1:B:73:ARG:CG	1:B:73:ARG:HH11	2.31	0.45
1:A:257:ALA:O	1:A:260:VAL:HG22	2.17	0.44
1:A:389:MET:O	1:A:389:MET:HG2	2.17	0.44
1:A:209:CYS:SG	1:A:210:ASN:N	2.91	0.44
1:B:311:ARG:O	1:B:314:VAL:HG12	2.17	0.44
1:A:-27:ARG:NH2	1:A:1:GLU:OE2	2.51	0.44
1:A:393:TRP:HA	1:A:393:TRP:CE3	2.52	0.44
1:B:233:GLN:HA	1:B:237:HIS:HB2	2.00	0.44
1:A:-110:LEU:HD11	1:B:347:ARG:HG2	2.00	0.44
1:A:333:TRP:CH2	1:A:337:LYS:HD2	2.53	0.44
1:A:373:HIS:N	1:A:373:HIS:ND1	2.66	0.43
1:A:58:ALA:HB1	1:B:347:ARG:NH1	2.33	0.43
1:B:281:ILE:HA	1:B:284:VAL:HG22	2.00	0.43
1:B:-97:ASP:OD2	1:B:-6:ASN:ND2	2.46	0.43
1:A:357:ASN:OD1	1:A:357:ASN:N	2.50	0.43
1:A:-103:PHE:HE2	1:A:-36:VAL:HG13	1.83	0.43
1:B:257:ALA:HA	1:B:260:VAL:HG22	1.99	0.43
1:A:263:LEU:HD23	1:A:263:LEU:O	2.18	0.43
1:A:340:LYS:HE3	1:B:-106:GLY:CA	2.48	0.43
1:A:-29:ILE:HD11	1:A:-7:ILE:HA	1.99	0.43
1:B:137:ASP:OD1	1:B:204:GLU:N	2.52	0.43
1:A:384:GLU:O	1:B:291:VAL:HG22	2.19	0.43
1:A:-15:ASP:O	1:A:-11:ARG:HG3	2.18	0.42
1:B:-9:ALA:O	1:B:-5:MET:HG3	2.19	0.42
1:A:380:VAL:O	1:A:380:VAL:CG1	2.67	0.42
1:B:-10:CYS:HB3	1:B:51:TRP:CH2	2.55	0.42

Continued on next page...

CONFIDENTIAL VALIDATION REPORT

Continued from previous page...

Atom-1	Atom-2	Interatomic distance (Å)	Clash overlap (Å)
1:B:343:GLU:O	1:B:347:ARG:HG3	2.19	0.42
1:B:242:TYR:CD1	1:B:242:TYR:O	2.72	0.42
1:A:-5:MET:O	1:A:-1:MET:HG2	2.19	0.42
1:B:-5:MET:SD	1:B:46:PHE:CE2	3.09	0.42
1:B:-100:LEU:HD13	1:B:-100:LEU:HA	1.92	0.42
1:B:-104:ILE:O	1:B:-104:ILE:HG13	2.20	0.42
1:A:-11:ARG:O	1:A:-7:ILE:HG13	2.20	0.42
1:A:302:ILE:HG13	1:A:303:MET:N	2.33	0.41
1:A:359:THR:HG23	1:B:227:LEU:HD12	2.01	0.41
1:A:389:MET:SD	1:A:389:MET:N	2.93	0.41
1:A:257:ALA:HA	1:A:260:VAL:HG22	2.02	0.41
1:A:278:ARG:O	1:A:281:ILE:HG12	2.20	0.41
1:B:16:GLN:O	1:B:17:LYS:HB2	2.20	0.41
1:A:277:VAL:O	1:A:281:ILE:HG23	2.21	0.41
1:B:-43:GLU:O	1:B:-39:ASN:ND2	2.45	0.41
1:A:208:TYR:HA	1:A:216:PRO:HA	2.03	0.41
1:A:-29:ILE:HG12	1:A:-4:VAL:HG21	2.03	0.41
1:B:-11:ARG:O	1:B:-7:ILE:HG13	2.21	0.40
1:A:129:ILE:O	1:A:129:ILE:HG13	2.20	0.40
1:A:359:THR:C	1:A:361:LEU:H	2.24	0.40
1:B:76:ARG:HB3	1:B:76:ARG:HE	1.69	0.40
1:A:383:VAL:O	1:B:405:PHE:N	2.54	0.40

There are no symmetry-related clashes.

5.3 Torsion angles (i)

5.3.1 Protein backbone (i)

In the following table, the Percentiles column shows the percent Ramachandran outliers of the chain as a percentile score with respect to all X-ray entries followed by that with respect to entries of similar resolution.

The Analysed column shows the number of residues for which the backbone conformation was analysed, and the total number of residues.

Mol	Chain	Analysed	Favoured	Allowed	Outliers	Percentiles	
1	A	433/544 (80%)	403 (93%)	28 (6%)	2 (0%)	31	65
1	B	435/544 (80%)	406 (93%)	28 (6%)	1 (0%)	49	81
All	All	868/1088 (80%)	809 (93%)	56 (6%)	3 (0%)	43	75

CONFIDENTIAL VALIDATION REPORT

All (3) Ramachandran outliers are listed below:

Mol	Chain	Res	Type
1	B	218	VAL
1	A	360	ALA
1	A	385	PRO

5.3.2 Protein sidechains ⓘ

In the following table, the Percentiles column shows the percent sidechain outliers of the chain as a percentile score with respect to all X-ray entries followed by that with respect to entries of similar resolution.

The Analysed column shows the number of residues for which the sidechain conformation was analysed, and the total number of residues.

Mol	Chain	Analysed	Rotameric	Outliers	Percentiles	
1	A	372/468 (80%)	345 (93%)	27 (7%)	15	41
1	B	374/468 (80%)	351 (94%)	23 (6%)	20	51
All	All	746/936 (80%)	696 (93%)	50 (7%)	18	46

All (50) residues with a non-rotameric sidechain are listed below:

Mol	Chain	Res	Type
1	A	-104	ILE
1	A	-103	PHE
1	A	-52	ASN
1	A	48	THR
1	A	111	ASP
1	A	114	VAL
1	A	115	VAL
1	A	116	ASP
1	A	121	LYS
1	A	134	PHE
1	A	303	MET
1	A	308	LYS
1	A	357	ASN
1	A	367	LEU
1	A	370	PHE
1	A	373	HIS
1	A	377	LYS
1	A	379	ILE
1	A	380	VAL
1	A	381	ASP

Continued on next page...

CONFIDENTIAL VALIDATION REPORT

Continued from previous page...

Mol	Chain	Res	Type
1	A	386	HIS
1	A	389	MET
1	A	391	PHE
1	A	393	TRP
1	A	394	ASP
1	A	395	LEU
1	A	400	LYS
1	B	-110	LEU
1	B	-103	PHE
1	B	-87	ASP
1	B	-47	LYS
1	B	7	PHE
1	B	75	GLU
1	B	76	ARG
1	B	77	GLU
1	B	114	VAL
1	B	127	LYS
1	B	137	ASP
1	B	204	GLU
1	B	230	ARG
1	B	241	GLN
1	B	242	TYR
1	B	278	ARG
1	B	341	HIS
1	B	351	TRP
1	B	370	PHE
1	B	373	HIS
1	B	391	PHE
1	B	392	GLN
1	B	399	THR

Some sidechains can be flipped to improve hydrogen bonding and reduce clashes. There are no such sidechains identified.

5.3.3 RNA ⓘ

There are no RNA molecules in this entry.

5.4 Non-standard residues in protein, DNA, RNA chains ⓘ

There are no non-standard protein/DNA/RNA residues in this entry.

CONFIDENTIAL VALIDATION REPORT

5.5 Carbohydrates (i)

There are no carbohydrates in this entry.

5.6 Ligand geometry (i)

Of 8 ligands modelled in this entry, 8 are monoatomic - leaving 0 for Mogul analysis.

There are no bond length outliers.

There are no bond angle outliers.

There are no chirality outliers.

There are no torsion outliers.

There are no ring outliers.

No monomer is involved in short contacts.

5.7 Other polymers (i)

There are no such residues in this entry.

5.8 Polymer linkage issues (i)

There are no chain breaks in this entry.

CONFIDENTIAL VALIDATION REPORT

6 Fit of model and data (i)

6.1 Protein, DNA and RNA chains (i)

In the following table, the column labelled '#RSRZ> 2' contains the number (and percentage) of RSRZ outliers, followed by percent RSRZ outliers for the chain as percentile scores relative to all X-ray entries and entries of similar resolution. The OWAB column contains the minimum, median, 95th percentile and maximum values of the occupancy-weighted average B-factor per residue. The column labelled 'Q< 0.9' lists the number of (and percentage) of residues with an average occupancy less than 0.9.

Mol	Chain	Analysed	<RSRZ>	#RSRZ>2			OWAB(Å^2)	Q<0.9
1	A	439/544 (80%)	0.24	10 (2%)	60	58	48, 106, 161, 242	1 (0%)
1	B	441/544 (81%)	0.22	7 (1%)	72	71	40, 112, 174, 375	0
All	All	880/1088 (80%)	0.23	17 (1%)	66	64	40, 108, 168, 375	1 (0%)

All (17) RSRZ outliers are listed below:

Mol	Chain	Res	Type	RSRZ
1	A	95	ALA	9.3
1	B	-28	LEU	3.6
1	A	140	SER	3.5
1	B	406	GLY	3.4
1	B	72	LEU	3.0
1	B	109	ALA	2.8
1	B	21	GLU	2.6
1	B	-25	ALA	2.5
1	A	-52	ASN	2.5
1	A	314	VAL	2.4
1	A	397	ALA	2.4
1	A	256	LEU	2.4
1	B	365	LYS	2.4
1	A	213	LYS	2.3
1	A	359	THR	2.2
1	A	-7	ILE	2.2
1	A	14	LEU	2.1

6.2 Non-standard residues in protein, DNA, RNA chains (i)

There are no non-standard protein/DNA/RNA residues in this entry.

CONFIDENTIAL VALIDATION REPORT

6.3 Carbohydrates (i)

There are no carbohydrates in this entry.

6.4 Ligands (i)

In the following table, the Atoms column lists the number of modelled atoms in the group and the number defined in the chemical component dictionary. The B-factors column lists the minimum, median, 95th percentile and maximum values of B factors of atoms in the group. The column labelled 'Q< 0.9' lists the number of atoms with occupancy less than 0.9.

Mol	Type	Chain	Res	Atoms	RSCC	RSR	B-factors(Å^2)	Q<0.9
2	ZN	B	501	1/1	0.81	0.21	140,140,140,140	0
2	ZN	B	504	1/1	0.89	0.36	247,247,247,247	0
2	ZN	A	502	1/1	0.90	0.20	132,132,132,132	0
2	ZN	B	502	1/1	0.92	0.10	262,262,262,262	0
2	ZN	A	503	1/1	0.96	0.11	342,342,342,342	0
2	ZN	A	501	1/1	0.98	0.22	61,61,61,61	0
2	ZN	A	500	1/1	0.98	0.18	79,79,79,79	0
2	ZN	B	503	1/1	1.00	0.20	70,70,70,70	0

6.5 Other polymers (i)

There are no such residues in this entry.

CONFIDENTIAL VALIDATION REPORT
